# Hypertension among adults in a deprived urban area of Peru – Undiagnosed and uncontrolled?

**DOI:** 10.1186/1756-0500-1-2

**Published:** 2008-02-26

**Authors:** Alisha R Davies, J Jaime Miranda, Robert H Gilman, Liam Smeeth

**Affiliations:** 1 Department of Epidemiology and Population Health, London School of Hygiene and Tropical Medicine, London, UK; 2 Facultad de Salud Pública y Administración, Universidad Peruana Cayetano Heredia, Lima, Peru; 3 Department of International Health, Johns Hopkins Bloomberg School of Public Health, Baltimore, MD, USA; 4 Laboratorios de Investigación y Desarrollo en Ciencia y Tecnología, Facultad de Ciencias y Filosofía, Universidad Peruana Cayetano Heredia, Lima, Peru

## Abstract

**Background:**

In Peru, cardiovascular disease was the second most common cause of death in those aged 65 years or more in 2000. Hypertension is a major modifiable risk factor for cardiovascular disease, and if treated can significantly reduce cardiovascular disease risk. The objectives of this study were to investigate the prevalence of hypertension and levels of awareness, treatment and control in a deprived urban area of Peru.

**Methods:**

A cross-sectional study was completed. Blood pressure measurements were recorded in triplicate. Hypertension was defined as systolic blood pressure ≥ 140 mmHg or diastolic blood pressure ≥ 90 mmHg, or self report of receiving antihypertensive medication at the time of interview.

**Results:**

The study sample was 584 adults (29.1% male, mean age 35.3 years). Age standardized prevalence of hypertension was 19.5% (95% CI 9.9%, 29.1%) in men, 11.4% (95% CI 3.7%, 19.1%) in women, and 13.2% (95% CI 5.0%, 21.5%) overall. Among those with hypertension 38.3% (95% CI 22.7%, 53.9%, n = 18/47) were aware of their condition with greater awareness among women than men. Of those aware, 61.1% (n = 11/18) were treated, equating to 23.4% (95% CI 10.1%, 36.7%, n = 11/47) of all adults with hypertension. Of those treated 63.6% (n = 7/11) had controlled hypertension, equating to 14.9% (95% CI 3.0%, 26.8%, n = 7/47) of all adults with hypertension.

**Conclusion:**

Levels of awareness and control in this population were low. Lack of control is likely to be due to both a failure to diagnose hypertension, especially among men, and initiate or comply with treatment, especially among women. These results suggest a considerable burden of undiagnosed hypertension, and poor levels of control in those treated, in a deprived urban area of Lima, Peru.

## Background

Non-communicable diseases (NCD) are emerging as a major concern for health in developing countries [1]. In 2000, two thirds of all deaths in Latin America and the Caribbean were due to NCDs, with cardiovascular disease (CVD) alone accounting for 31% of deaths [2]. In Peru, CVD was the second most common cause of death in those aged 65 years or more (mortality rate 4 per 1000 population) in 2000. Ischaemic heart disease, heart failure and hypertension are also among the ten most common causes of death reported in this age group [3]. The increasing importance of cardiovascular disease in Latin American has been commented on [2,4,5] and the need for preventative action to be taken to limit, or prevent, a predicted epidemic of cardiovascular disease has been highlighted [6].

Hypertension is a major modifiable risk factor for CVD, and if treated can significantly reduce the risk of CVD. Despite this, many countries, including Europe, America and South Africa, have reported a high proportion of adults with hypertension remain unaware of their condition and fail to be treated [7-11]. Studies investigating hypertension in Peru are scant, with estimates of the prevalence of hypertension range from 16% to 31% in men, and 15% to 23% in women depending on the population studied [12-15]. In some regions of Peru it has been found that approximately 50% of adults with hypertension are unaware of their condition and in many their condition is poorly controlled [12,14]. In this study, we report the prevalence of hypertension in adults in a deprived urban area of Lima, Peru, and provide information on the diagnosis and treatment of hypertension among this population.

## Methods

In 2001, newborn children were enrolled in a cohort study established for diarrhoeal disease surveillance in San Juan de Miraflores, a deprived urban area of Lima, Peru. This cohort followed similar methodologies to previous diarrhoeal studies carried out by our research group in the same area before [16,17]. Enrolled children acted as the index children from which adult family members were invited to participate in a cross-sectional study on hypertension, the results of which are presented in this paper. In 2006, 497 index children were revisited and adult (aged >17 years) family members living in the same house as each child were invited to participate in the hypertension study. Informed consent was obtained. No information was recorded on adults who declined to participate. Participating adults were asked if they had ever had a medical diagnosis of hypertension, and if they were prescribed anti-hypertensive medications. Age, height and weight were recorded at interview and the body mass index (BMI) calculated (kilogram per metre^2^). BMI was classified as not overweight (BMI<25), overweight (25 ≤ BMI <30) or obese (BMI ≥ 30). Household bedroom ratio (number of household residents per bedroom) and number of years of education were obtained from the diarrhoeal cohort dataset. Blood pressure (BP) was measured using appropriate cuffs for arm circumference and measurements recorded in triplicate. The BP mean of the last two systolic and diastolic measurements were used in the analysis. Hypertension was defined as systolic blood pressure (SBP) ≥ 140 mmHg or diastolic blood pressure (DBP) ≥ 90 mmHg [18,19], or self report of receiving antihypertensive medication at the time of interview. Prevalence is the proportion of adults with hypertension and presented as a percentage. Direct standardisation was used to calculate age-standardized prevalence of hypertension by applying age group-specific prevalence (five yearly age groups: 20–24, 25–29 up to 70+ years) to the World Health Organization standard population [20]. Differences between those with hypertension and without were compared using logistic regression and odds ratios (OR) and their 95% confidence intervals (CI) are presented. Multiple adults were recruited within a single family unit, and so all analyses were adjusted for the clustering at family unit level. Awareness was defined as the proportion of hypertensive adults reporting a prior medical diagnosis of hypertension or high BP. Controlled hypertension was defined as SBP < 140 mm Hg and DBP < 90 mmHg. Ethical approval was granted by the Universidad Peruana Cayetano Heredia ethics committee.

## Results

A total of 584 adults agreed to participate, representing 67.8% of the families visited (337/497). Only one adult was recruited from 166 family units, and multiple adults were recruited from the remaining 331 families (average number of adults per family unit 1.3 (range 2 to 10)). The majority of the participants (61.0%) were parents of the children recruited in the cohort.

Of the 584 (29.1% male) adults interviewed the mean age was 35.3 years (95% CI 34.4, 36.3). The mean age was 36.7 (95% CI 34.8, 38.7) years in men and 34.8 (95% CI 33.7, 35.8) years in women. Characteristics of the study population are provided in Table 1. Information on BMI was available for 579 adults, 42.1% of whom were overweight, and 18.2% obese. A greater proportion of women were obese compared to men (F statistic = 6.4, p = 0.002). The majority of adults had some level of education, but a greater proportion of men reported levels of secondary education (67.1%) compared to women (49.0%).

**Table 1:** Baseline descriptive information, mean and standard error diastolic and systolic blood pressure measurements and hypertension prevalence among adults in San Juan de Miraflores region of Lima, Peru, 2006.

	Men (n = 170)	Women (n = 414)	p value	Total (n = 584)
*Age group (n, %)*			0.161^1^	
<30 years	49 (28.8)	146 (35.3)		195 (33.4)
30–49 years	103 (60.6)	237 (57.3)		340 (58.2)
50+ years	18 (10.6)	31 (7.5)		49 (8.4)
*BMI (n, %)*			0.002^1^	
Not overweight	78 (45.9)	149 (36.0)		227 (38.9)
Overweight	73 (42.9)	173 (41.8)		246 (42.1)
Obese	16 (9.4)	90 (21.7)		106 (18.2)
Missing	3 (1.8)	2 (0.5)		5 (0.9)
*Household bedroom ratio (quartiles)*			0.021^1^	
1 (lowest)	38 (22.4)	81 (19.6)		119 (20.4)
2	29 (17.1)	77 (18.6)		106 (18.2)
3	29 (17.1)	91 (22.0)		120 (20.6)
4 (highest)	65 (38.2)	113 (27.3)		178 (30.5)
Missing	9 (5.3)	52 (12.6)		61 (10.5)
*Number of years of education*			0.005^1^	
0	1 (0.6)	6 (1.5)		7 (1.2)
1 to 6	27 (15.9)	101 (24.4)		128 (21.9)
7 to 11	114 (67.1)	203 (49.0)		317 (54.3)
12 to 21	13 (7.7)	44 (10.6)		57 (9.8)
Missing	15 (8.8)	60 (14.5)		75 (12.8)
*Systolic blood pressure *(mmHg, SE)	119.3 (1.0)	109.5 (0.7)	<0.001^2^	
*Diastolic blood pressure *(mmHg, SE)	74.9 (0.8)	68.3 (0.6)	<0.001^2^	
*Prevalence of hypertension *(%, 95% CI)				
<30 years	0	2.7 (0.1, 5.4)	0.233^1^	2.1 (0, 4.1)
30–49 years	14.6 (7.6, 21.5)	5.9 (2.9, 8.9)	0.008^1^	8.5 (5.5, 11.6)
50+ years	27.8 (6.0, 49.5)	29.0 (12.1, 46.0)	0.927^1^	28.6 (16.2, 41.0)
Crude (all ages)	11.8 (6.9, 16.7)	6.5 (4.1, 8.9)	0.036^1^	8.0 (5.8, 10.3)
Age standardized^3 ^(20 to 70+ years)	19.5 (9.9, 29.1)	11.4 (3.7, 19.1)	0.674^2^	13.2 (5.0, 21.5)

^1^cluster design based p value from F statistic; ^2^z test; ^3^Standardized to the World Health Organization world population standard; SE standard error; CI confidence interval.

The mean SBP was 119.3 mmHg (SE 1.0) in men and 109.5 mmHg (SE 0.7) in women. The mean DBP was 74.9 mmHg (SE 0.8) in men and 68.2 mmHg (SE 0.6) in women (Table 1). A positive correlation between BP and age was found, with a single year increase in age equating to a 0.5 mmHg (95% CI 0.4, 0.6, p < 0.001) increase in SBP and a 0.2 mmHg (95% CI 0.1, 0.3, p < 0.001) increase in DBP. Forty-seven adults had evidence of hypertension, equating to a crude prevalence of 8.0% (95% CI 5.8%, 10.3%) in the study population. Hypertension was more prevalent in men compared with women, and increased with increasing age group (Table 1). Age standardized prevalence of hypertension was 13.2% (95% CI 5.0%, 21.5%), and greater in men (19.5% (95% CI 9.9%, 29.1%)) than women (11.4% (95% CI 3.7%, 19.1%)). Among those with untreated hypertension the predominant type of hypertension was isolated diastolic hypertension (42.5%, 17/40) compared to isolated systolic hypertension (27.5%, 11/40) and systodiastolic hypertension (30.0%, 12/40).

The crude odds of hypertension in men were twice that found in women (Table 2), but after adjusting for explanatory factors in the multivariate analysis no evidence of a sex difference in the prevalence of hypertension remained. In the crude logistic regression there was some evidence to suggest that the odds of hypertension were greater in those overweight or obese, and in those with no education, however these associations were not found in the multivariate analysis (Table 2). There was strong evidence of an increasing odds of hypertension with increasing age, and this remained after adjustment for other explanatory factors (Table 2).

**Table 2:** Crude and adjusted odds ratio for risk factors for hypertension among adults in San Juan de Miraflores region of Lima, Peru, 2006.

	Crude		Adjusted^1 ^(n = 505)	
		
	Odds ratio (95% CI)	p value	Odds ratio (95% CI)	p value
*Sex*				
Female	1		1	
Male	1.9 (1.0, 3.5)	0.038	1.7 (0.8, 3.3)	0.135
*Age group*				
>30 years	1		1	
30–49 years	4.5 (1.6, 12.7)	0.005	4.5 (1.3, 15.4)	0.018
50+ years	19.1 (6.1, 60.2)	<0.001	18.9 (4.9, 73.1)	<0.001
*BMI*				
Not overweight	1		1	
Overweight	2.5 (1.2, 5.2)	0.018	1.9 (0.8, 4.7)	0.138
Obese	2.3 (0.9, 5.7)	0.085	2.2 (0.8, 6.1)	0.133
*Household bedroom ratio (quartiles)*				
1 (lowest)	1		1	
2	0.7 (0.3, 1.7)	0.439	1.1 (0.4, 2.6)	0.880
3	0.5 (0.2, 1.2)	0.102	0.7 (0.2, 1.7)	0.395
4 (highest)	0.4 (0.2, 1.0)	0.049	0.6 (0.2, 1.4)	0.222
*Number of years of education*				
0	11.0 (1.2, 98.4)	0.032	1.1 (0.1, 21.7)	0.936
1 to 6	2.6 (0.6, 12.4)	0.234	1.8 (0.3, 9.4)	0.499
7 to 11	2.5 (0.5, 11.0)	0.238	2.3 (0.5, 11.3)	0.311
12 to 21	1		1	

^1 ^adjusted for all other variables listed in the table; CI confidence interval. Adjusted for clustering.

**Figure 1:**
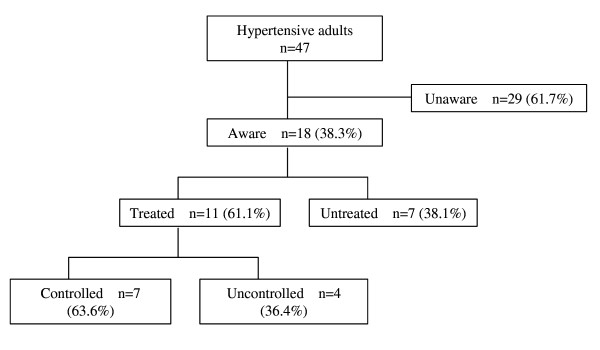
The proportion awareness, treatment and control among hypertensive adults in San Juan de Miraflores region of Lima, Peru, 2006.

Only one third (38.3% (95% CI 22.7%, 53.9%)) of all hypertensive adults were aware of their condition (Figure 1). There was evidence to suggest a greater proportion of women (55.6% (95% CI 34.9%, 76.2%), n = 15/27) were aware of their hypertension, compared to men (15.0% (95% CI -2.1%, 32.1%), n = 3/20) (F statistic = 8.5, p value = 0.006). A trend of increasing awareness with decreasing household bedroom ratio, an indicator of socio-economic status, was found (F statistic = 2.8, p = 0.042). There was no evidence to suggest a difference in awareness with age (F statistic = 0.9, p = 0.419), and no evidence of greater awareness in the obese compared to the non-obese (F statistic = 3.1, p = 0.084). Among adults aware of their hypertension 61.1% (95% CI 36.3%, 85.9%, n = 11/18) reported receiving anti-hypertensive medication, equating to 23.4% (95% CI 10.1%, 36.7%, n = 11/47) of all adults with hypertension (Figure 1). Although the numbers are small there was some evidence of a sex difference in the treatment of hypertension, with only 53.3% (95% CI 26.4%, 80.2%, n = 8/15) of the women aware of their hypertension receiving treatment, compared to all of the men (100%, n = 3/3). Among treated, 63.6% (95% CI 27.3%, 100.0%, n = 7/11) had controlled hypertension, equating to only 14.9% (95% CI 3.0%, 26.8%, n = 7/47) of all adults with hypertension.

## Discussion

In our study, we report the age standardised prevalence of hypertension among young adults in a deprived urban area of Lima was 13.2% (95% CI 5.0%, 21.5%). Overall, 38% (95% CI 22.7%, 53.9%) of all adults with hypertension were aware of their diagnosis, 23% (95% CI 10.1%, 36.7%) were receiving treatment, and control was achieved in only 15% (95% CI 3.0%, 26.8%) of hypertensive adults.

### Comparison with other studies

The crude prevalence of hypertension found in this study (8.0% (95% CI 5.8%, 10.3%)) was lower than that reported by other studies in Peruvian populations and this is likely to reflect the young age of our study population [12-15]. A cross-sectional study of the adult population in Peru by the Peruvian Society of Cardiology reported a national prevalence of 23.7% (men 27.1%, women 20.4%), but prevalence was lower (15.3%) in the south of Lima, the geographical region most comparable to our study [12]. More robust estimates of prevalence are available from the PREVENCION study carried out in a large city in the mountainous regions of Peru. The prevalence of hypertension found among a representative population of adults with an older mean age that our study population (49.1 compared to 35.3 years) was 15.7% (95% CI 14.0 to 17.4%) [14].

### Awareness

We found that 38% of adults with hypertension were aware of their condition, and awareness was much greater in women compared to men. Similar levels of awareness, and supporting evidence of a sex difference in awareness, have been reported by other studies in Peru [12,14]. Awareness in Peru is low compared to international estimates, for example a study in Sao Paulo, Brazil, reported three quarters of those with hypertension were aware of their condition [21]. A review of hypertension in national representative population surveys completed in the 1990s reported the awareness among the adult (35 to 64 years) population was 69% in America, 63% in Canada, and ranged from 36 to 52% in five European countries [11,22].

### Controlled hypertension

In our study population only 23% of those with hypertension were receiving pharmaceutical treatment and hypertension control was achieved in two thirds of those treated. These estimates are comparable to that reported by the PREVENCION study (39.5% treated, 35.7% controlled among those treated) [14] and the national survey (28.1% treated, 23.6% controlled among those treated) [12]. Treatment levels in Peru are much lower than in America (53%), Canada (36%), Europe (25 to 60%) and Brazil (62%) [11,21-23]. The lower treatment rates found in Peru may be due to social and cultural differences, for example adults tend to attend a doctor or take medication only if they feel ill. This may result in low diagnosis rates and reduce the opportunity to provide treatment and advice on the importance of compliance. In addition, there are economic factors to consider as adults must pay for prescribed medications in Peru.

Among those aware of their hypertension we found evidence to suggest women were less likely to be treated than men. This may be due to the small numbers investigated, as no sex difference in treatment was found in the PREVENCION study [14]. However, it may also reflect gender differences in access to treatment despite being aware of their condition. Further investigation is needed to determine if there is a true sex difference in treatment among this poor population, and if so, to explore the possible underlying reasons. Encouragingly we found hypertension was controlled in two thirds of the adults treated, which is comparable to other developed countries. However, this equates to achieving control in only 15% of all adults with hypertension, which is comparable to that reported in other areas of Peru [12,14]. Lack of control among our study population is likely to be due to both a failure to diagnose hypertension, especially among men, and initiate or comply with treatment, especially among women. Over time, as the patient ages, uncontrolled hypertension can result in more severe cardiovascular problems. To find this level of uncontrolled hypertension among a relatively young population is of cause for concern, as the detrimental effects on health will increase as the population ages.

### Strengths and weaknesses

This is a descriptive observational study and, as such, has a number of limitations. A possible sampling bias, due to the selection of adults from families who had already agreed to their child participating in a diarrhoeal follow up cohort, could be expected. However, the initial birth cohort was randomly selected and as being born in a community is a random process, and family members of each child were invited to participate based on these index children, the adults in this hypertension study may be considered a quasi-random sample of adults from the area of San Juan de Miraflores, Lima, Peru. Only a small number of patients with hypertension were identified, and as a result of the small sample size the estimates of hypertension awareness, treatment and control may not be robust and should be considered with caution. A further limitation of the small study sample was that men were under-represented, accounting for less than one third of the adults interviewed, and as a result the confidence interval for the estimate of hypertension prevalence among men was wide. The aim of our study was not to investigate sex differences in awareness, treatment and control, and therefore such findings should be treated with caution. The study design meant that some individuals were recruited from the same family units, or clusters. One consideration of this type of data is that individuals recruited from the same family units (or clusters) are likely to be more similar to each other than to individuals from different families. However, the extent of clustering was minimal (one adult was recruited from 166 family units, and in the remaining 331 family units (clusters) the average number of adults recruited from each cluster was 1.3). Further adjustment for clustering did not affected the point estimates of prevalence and treatment and awareness, and only had a limited effect on the confidence intervals, e.g. the crude prevalence of hypertension unadjusted for clustering was 8.0% (95% CI 5.8%, 10.3%) and after adjustment for clustering the confidence interval remained 5.8% to 10.3% due to the very small change to the standard error. A further limitation of this study is that the estimates of awareness are subject to recall bias, as an individual's awareness of hypertension was based on self-report. Finally, although the BP measurements were completed in triplicate, they were taken only on one day and it is recommended that a diagnosis of hypertension in adults be made by multiple BP measurements recorded on separate occasions [19].

## Conclusion

Hypertension is a major modifiable risk factor and if left uncontrolled it can result in major cardiovascular problems in later life.Although this study is small and has a number of limitations it does raise some important questions about the provision of care and treatment for patients with hypertension in a deprived urban area of Peru. The results, along with that from other published studies indicate a large burden of undiagnosed hypertension in the population of Peru, and poor levels of control. This set within the context a country with underlying difficulties in the provision of health care represents a great challenge for public health in Peru.

## Competing interests

The authors declare that they have no competing interests.

## Authors' contributions

JJM conceived the study design, was responsible for the data collection, contributed to the data analysis and writing the paper. ARD completed analyses, interpreted findings, and was the principal author of the manuscript. RHG and LS were involved in the study design, and contributed to the interpretation of data, and editing the paper.
